# Designing Supportive Soundscapes for Nursing Home Residents with Dementia

**DOI:** 10.3390/ijerph16244904

**Published:** 2019-12-04

**Authors:** Paul Devos, Francesco Aletta, Pieter Thomas, Mirko Petrovic, Tara Vander Mynsbrugge, Dominique Van de Velde, Patricia De Vriendt, Dick Botteldooren

**Affiliations:** 1 Department of Information Technology, Ghent University, 9052 Ghent, Belgium; f.aletta@ucl.ac.uk (F.A.); pieter.thomas@ugent.be (P.T.); Dick.Botteldooren@UGent.be (D.B.); 2 Institute for Environmental Design and Engineering, University College London, London WC1H0NN, UK; 3 Department of Internal Medicine and Paediatrics, Ghent University, 9000 Ghent, Belgium; Mirko.Petrovic@UGent.be; 4 Department of Occupational Therapy, Artevelde University College, 9000 Ghent, Belgium; tara.vandermynsbrugge@arteveldehs.be (T.V.M.); dominique.vandevelde@arteveldehs.be (D.V.d.V.); Patricia.DeVriendt@arteveldehs.be (P.D.V.); 5 Department of Occupational Therapy, Ghent University, 9000 Ghent, Belgium

**Keywords:** supportive soundscape, sonic environment, nursing homes, ageing, dementia

## Abstract

Sound and its resulting soundscape is a major appraisal component of the living environment. Where environmental sounds (e.g., outdoor traffic sounds) are often perceived as negative, a soundscape (e.g., containing natural sounds) can also have a positive effect on health and well-being. This supportive effect of a soundscape is getting increasing attention for use in practice. This paper addresses the design of a supportive sonic environment for persons with dementia in nursing homes. Starting from a review of key mechanisms related to sonic perception, cognitive deficits and related behavior, a framework is derived for the composition of a sonic environment for persons with dementia. The proposed framework is centered around using acoustic stimuli for influencing mood, stimulating the feeling of safety and triggering a response in a person. These stimuli are intended to be deployed as added sounds in a nursing home to improve the well-being and behavior of the residents.

## 1. Introduction

As ageing is a dominant concern of today’s society, adopting health care towards the needs of older people is an important challenge. With regard to ageing, one of the major causes of disability and dependency among older people is dementia. Dementia is a syndrome in which abnormal cognitive impairment leads to disability and dependency. It is the additional deterioration of cognitive capabilities compared to normal ageing deterioration. It originates from underlying disease induced brain changes and results in impairment of memory, thinking, orientation, awareness, comprehension, calculation, learning capacity, language and judgment [1]. As a consequence daily functioning is hindered. Dementia can also result in challenging behavior, leading to a variety of Behavioral and Psychological Symptoms of Dementia (BPSD). Different underlying diseases like prion disease, Alzheimer disease, vascular dementia, fronto-temporal dementia (e.g., semantic dementia), Parkinsons’ disease, dementia with Lewy bodies and others are known to result in dementia, with 47 million people affected worldwide and a prevalence of 10 million affected people each year [2,3].

In order to have permanent care guaranteed from accessible and supportive care givers, people with dementia can reside in nursing homes. Such institutions are operating to provide residential accommodation with supervision from nursing staff 24 h a day, meals, help with personal care needs and additional specialized services to older people. The residents occupy a sleeping room and can reside during specific daytime periods in a living room, where social interaction and group support can take place. As the behavior of a person is related to his well-being, the underlying determinants like health, environment and social activity are important aspects for the delivery of a high quality of life in these accommodations. In order to guarantee a high quality of life for the residents and especially in the case of residents with dementia the provided care should span medical, social and supportive care [4].

There is a growing understanding of how various aspects of the living environment could affect health and well-being, in particular for persons with reduced mental capabilities. Directly, as well as indirectly, this also affects care professionals. Such understanding is needed in the design of healing environments [5].

An important component of the living environment is the sonic environment, which, in combination with the perception of it by a person or a group of persons in a specific context, is known as a soundscape. A sonic environment and a soundscape are related but they are not quite the same thing. The former refers to the collection of physical sounds present and audible in a given space, while the latter is the perceptual construct resulting for a person exposed to this sonic environment. The ISO working group recently clarified this issue by defining the soundscape as an “acoustic environment as perceived or experienced and/or understood by a person or people, in context” [6]. Thus, it is essential to understand what people actually “perceive” instead of merely measuring the physical properties of a sonic environment. The context considered here is a typical nursing home, which is a medicalized, institutional care and living facility context.

By providing people with signals about the environment they experience, sound plays a crucial role as it can influence cognition and thus, also behavior [7]. Many studies dealing with the treatment of persons with dementia were underpinned by sensory stimulation but often failed to properly consider the everyday sonic environment and its potential to influence persons either positively or negatively [8]. Nevertheless, the auditory domain should be carefully taken into account for persons with dementia, since they are likely to rely more on sound than other people, due to a high prevalence of visual impairments compared to hearing impairments [9]. For this group of people, indeed, sound is often the pathway to making sense of the surrounding world, because with the impairment of one sense (e.g., vision), the auditory information can compensate for the negative effects of the degraded visual one. Providing conditions that not only “permit,” but rather “promote” supportive sonic environments could be beneficial for the well-being and quality of life of persons with dementia in care facilities. For this to happen, it is also necessary to raise awareness on this matter among the staff members working in the care sector [10,11]. Because of their “implicit knowledge about the role of the auditory environment into the daily practice of working with persons with an intellectual disability” [9], they may play a crucial role in improving this environment through changes in modus operandi, a knowledge gained in the group of people with intellectual disability and also applicable to people with dementia.

In this paper, a framework for improving the auditory environment by adding acoustic stimuli to an existing acoustic environment in order to obtain supportive effects for persons with dementia, is proposed. The paper starts with a narrative review of elements contributing in the interplay between perception, cognition and behavior. This leads to the main result, the definition of targeted effects which can be understood as contributing to behavior influencers and its presentation in a framework for soundscape design in nursing homes. This framework is then discussed in view of general aspects and in view of related studies where soundscape deployment in nursing homes has been experimented.

## 2. State of the Art

The design of a sonic environment in the context of a nursing home hosting persons with dementia requires taking into account the existing evidence in the field of auditory perception and related behavior with respect to ageing and cognition deficits, the state of the art of which is narratively reviewed here.

### 2.1. Perception: From Sonic Environment (Acoustic Scene) to Soundscape

**Figure 1:**
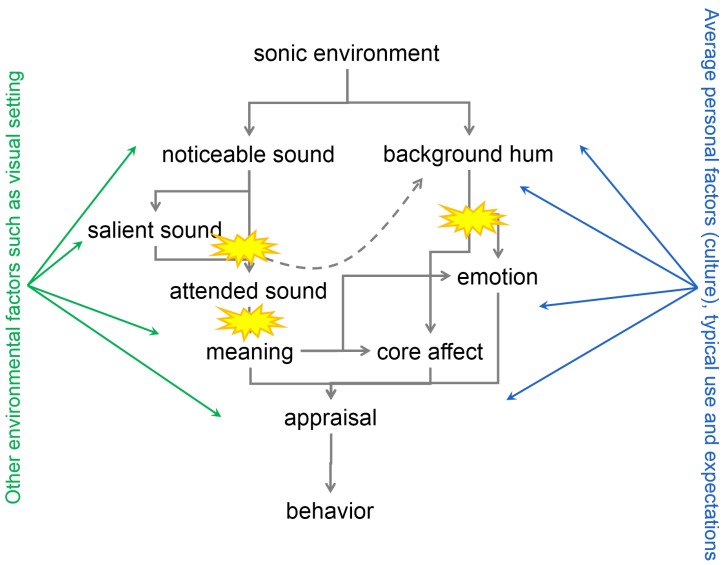
Human perception model: from sonic environment to soundscape appraisal (adapted from [21]). The surprise text balloons indicate some possible deficits resulting in e.g., deviant appraisal (upper left): impaired perception of sound features; (lower left): impaired recognition of sounds; (right): impaired perception of auditory scenes and objects.

Having defined the difference between a sonic environment and a soundscape, it is important to understand what makes the construction of the latter possible, descending from the former. Identifying how the mechanisms involved might be different for people with cognitive impairments will allow us to derive the design methodology. Due to the ubiquitous nature of sound, the scanning of all sounds present in a sonic environment demands a very high cognitive load, making a saliency mechanism in sound perception beneficial [12]. Auditory attention plays a key role in this process [13]. The ability of a sound to attract attention is in turn affected by a number of factors related to both the characteristics of the sound itself, as well as by personal traits of the listener, reflecting a bottom-up and top-down modulation of the attention mechanism. Attention can be modulated by ’bottom-up’ stimulus-driven factors (e.g., a loud explosion sound), ’top-down’ task-specific goals (e.g., in case of an announcement in a busy train station), expectations and learned schemas [14]. Although general attention is a multi domain modality making it necessary to account for multi sensory (visual) integration [15], the auditory attention mechanism remains in case of visual impairment. Sounds that are foregrounded by the attention and gating mechanisms will trigger associations. These are often related to sound sources or activities [16]. In a more general sense, these sounds have meaning. A simple interpretation of “meaning” could indeed be the collection of associations triggered by noticing a sound. Meaning is personal, but also has a cultural component. It can change over time through new experiences resulting in novel neural associations provided the plasticity of the brain is intact. The sonic environment, as a whole, also has the potential of creating meaning, changing mood, affect and emotion, irrespectively of whether it is split in separate auditory objects that receive attention. Music is by far the best-known example [17] but also natural and environmental sound environments may have music-like characteristics [18]. But even simple sounds can trigger an emotional response depending on their loudness and sharpness [19]. Appraisal of the soundscape involves the cognitive and emotional response described above related to personal expectations and frame of reference. Expectations in a shorter time frame influence the appraisal of the sonic environment [20], with moderate expectation violation creating the most pleasing environments. Liking and pleasure follow an inverse U curve with the degree of complexity or predictability of the environment (the Wundt curve). Very simple sonic environments, or cognitive and emotional journeys, are easily predicted and do not open up the possibility for learning. Very high complexity causes unpredictability and constant expectation violation, which also results in lower appraisal of the environment—the middle is just right. As experience grows, the inverse U curve shifts to higher complexities. A qualitative model relating sonic environment to soundscape is sketched in Figure 1 from Reference [21].

### 2.2. Perceived Safety Theory

Following Maslow’s hierarchy of needs, aside from essential physiological needs, safety is a basic need a person should fulfill [22]. Modern theories of perceived safety, such as the Generalized Unsafety Theory of Stress (GUTS), explain observations by assuming that the stress response is the default state that constantly needs to be inhibited [23,24]. Common situations where this is affecting behavior includes loneliness, low social status, adult life after prenatal or early life adversity, lack of a natural environment and less fit bodily states such as obesity or fatigue. Reflected to the context of nursing home residents with dementia, one could consider loneliness and lack of a natural environment as important situations. The absence of (environmental) signals that confirm safety may lead to chronic stress responses rather than the presence of instantaneous stressors. In this way, understanding where one is and understanding what time it is, will result in a behavior adapted to place and time. Since audition is the primary sense for detecting danger, it can be expected that the sonic environment has the power to influence perceived safety. Shäfer et al. [25] have shown the importance of stress and danger as perceived from music, silence and natural sounds (chirping crickets) in order to obtain indications of the environment, indicating that suitable music could be beneficial for this purpose.

### 2.3. Effects of Subliminal and Attended Sound on Behavior

The polarity of a sound with regard to its attended versus subliminal nature is giving two distinct ways to influence the behavior of a person. In a care setting where the use of sounds is considered, music therapy is an example of the behavior influencing potential of attended sounds. Music has been shown to give a manifold of positive effects, ranging from more physiological effects (arouse body temperature, reduce muscle tension, lower blood pressure, enhance depth breathing, elevate brain waves) to emotional or functional effects (influence emotion, decrease depression/improve mood, increase endurance and productivity, decrease anger, improve memory and learning, enhance sleep quality) [26,27]. Considering the emotional response, music is able to influence the mood and the emotions of a person and the ‘Musical Mood Induction Procedure’ (MMIP) [17] has gained a lot of attention and has shown that combined specific characteristics of the different musical elements (mode, tempo, pitch, rhythm, harmony and loudness) give rise to a range of emotional expressions (serious, sad, fear, serene, humorous, happy, exciting and majestic). In general, a slow tempo, low pitch and minor mode are associated with negative arousal and fast tempo, high pitch and major mode are associated with positive arousal. While music has a direct effect on mood its cognitive processing can also remind people of valued past events [28]. The initial mood is of importance in obtaining a high level of desired mood. Personal factors need to be taken into account, since individual differences occur from musical experience, preferences and traits. Indeed, it is important to state that music has a potential twofold outcome, as it can result in a positive or negative impact [26]. Apart from music, many different sounds can be present and take part in an acoustic scene. In general they can be classified following a detailed taxonomy of possible sound sources in a specific context (e.g., References [29,30]). A primary classification lies in the distinction between biophonic, geophonic and anthropogenic sounds. Nature and an associated natural sound environment have been shown to increase mental restoration after stressful periods [31] and the facilitation of mood recovery [32]. As components of nature, in a restorative environment one can consider ’being away’ (giving rest in directed attention), fascination (freedom of thinking), extent (freedom) and compatibility (resulting in facilitating efforts) [33]. Bird sounds and birdsong in particular may contribute to a positive feeling, perceived restoration of attention and stress recovery [34,35,36,37]. For most people, natural sounds and birdsong in particular create a positive valence and are perceived as calm or vibrant [38].

### 2.4. Changing Auditory Processing and Cognition with Age

Age-related hearing loss (Presbycusis) is characterized by reduced hearing sensitivity (higher frequencies) and speech understanding in noisy environments, slowed central processing of acoustic information, and impaired localization of sound sources [39]. This disorder affects hearing in more than half of the elderly population and is known to influence the peripheral and cortical auditory processing. In older adults, the interplay between these processing centers leads to a more dominant distractability due to decreased afferent information regulation [40]. The resulting deficits are mainly in sound localization and temporal processing, which lead to poor speech perception. It has been shown that age-related deficits in the interhemispheric information processing may be at the origin of different hearing problems among the older people [41]. As for temporal origin, older people have a degraded hearing gap detection, which leads to missing elements in the segmental information of speech and results in degraded speech perception in noise [42]. Apart from the degeneration of the auditory system due to the ageing process itself, noise damage also takes part as a dominant factor. In addition, genetic susceptibility, otological disorders and the use of ototoxic drugs (like aminoglycosides, quinine, bèta blockers, non-steroidal anti-inflammatory agents and tricyclic antidepressants) can contribute to the decline in hearing [39,43]. Accumulated drug intake is the case in a significant group of older people [43]. As part of the binaural localization, the source position estimation results from processing inter-aural intensity differences and inter-aural time differences, which are affected by hearing loss [44]. In addition, the auditory distance perception in humans is based on sound level, degree of reverberation and frequency as primary cues and on non perceptual factors, including the importance of the auditory event to the listener [45,46]. The degree of reverberation (direct-to-reverberant energy ratio) is of obvious importance in an indoor context. In case of hearing loss, the use of sound level as a distance cue remains effective, the use of the degree of reverberation as distance cue becomes less effective [46]. It was evidenced that brain regions for sound localization and for sound identification processing are distinct [47], as many of these auditory deficits reflect the deteriorated activity of specific cortex regions. Hearing impairment has negative impacts on quality of life and daily functioning for older persons, as they affect conversation, music appreciation, orientation to alarms and participation in social activities [39,48] as was shown in an longitudinal study of more than 2500 subjects [49]. Although hearing aids and assistive devices can have a positive impact, hearing aid uptake in older adults remains low despite the significant technological progress in hearing aid technology over the last decade [50].

### 2.5. Deviant Auditory Processing and Resulting Behavior in Dementia

Symptoms of altered auditory cognition due to dementia induced brain changes were studied in a clinically oriented symptom-based approach by Hardy et al. [51], showing that these symptoms range from impaired perception of sound features to impaired higher cognitive tasks as the recognition of sounds, auditory scenes and objects:

- Impaired perception of sound features: this may manifest as cortical deafness or relatively selective ‘word deafness’ or auditory agnosia, more commonly described with progressive non fluent aphasia.
- Impaired recognition of sounds: due to erosion of semantic memory deficits of nonverbal sound recognition (auditory associative agnosia) like the recognition of environmental sounds are present in patients with semantic dementia, while for some individuals recognition of melodies preserves.
- Impaired perception of auditory scenes and objects: in this case difficulty following conversations and other sounds against background noise are reported, this may result in avoiding social interactions and a general dislike of complex auditory environments.
- Auditory hallucinations: Tinnitus as an elementary auditory hallucination is commonly reported by patients with semantic dementia. Muffled sounds or voices as hallucinations are often reported by patients with Lewy body dementia, as well as other musical hallucinations (comprising persistent familiar, basal tunes).
- Abnormal auditory behaviors: In this case, deviant emotional or hedonic behavioral responses to sound are observed in patients with dementia (due to impaired recognition of musical and nonverbal vocal emotions). Sound aversion is present in many patients of fronto-temporal dementia. On the other hand, abnormal craving for music (musicophilia) is associated with semantic dementia, these patients may show increased sensitivity to sound (hyperacusis).

Where speech and music are main sources when considering sound, several studies provide information and results related to the cognition of nonverbal sounds in dementia [52,53,54]. As reported, the findings provide evidence that separable stages of auditory object analysis and separable profiles of impaired auditory object cognition can be considered as they are encountered in different dementia syndromes. From these studies, it is clear that the wide spectrum of dementia results in a wide scale and levels of deviation in the “sound-cognition-behavior” interplay (as illustrated in Figure 1), and that from the underlying disease, the typical characteristics of the interplay can be considered, resulting in more or less distinct groups among the persons with dementia.

## 3. Soundscape Design Framework

**Figure 2:**
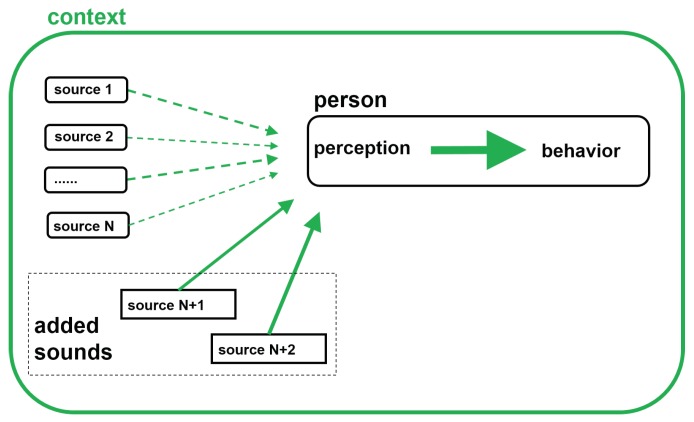
Schematic representation of the soundscape intervention introducing the added composed soundscape targeted to the desired behavioral response.

In view of the above mentioned successes of music therapy and in view of the stated effects that the sonic environment may have on people, the suggestion is to carefully design the sonic environment in the place of residence of persons with dementia. In contrast to their peers in the same age category, these persons no longer have the ability to participate in events nor to create their own comfortable and/or stimulating environment. The main difference between soundscape design and music therapy lies in the continuous character of the sonic environment. To this end, a framework for designing a soundscape for persons with dementia is proposed that is based on the above narrative review and that is intended to be used in a soundscape intervention as is indicated in Figure 2.

As previously described, it is clear that, due to dementia, different cognitive deficits can occur and can result in a perturbed sound perception, interpretation, appraisal and resulting behavior. While dementia is a syndrome of a wide window of cognitive deficits, it is a practical and attractive methodology to work with a limited number of distinguishable personas, which can identify the different over-all capabilities of the persons. In a first approach, a bi-level classification in interpreting and reacting capabilities of a person leads to an approach based on four personas [55,56]. Persons with a light form of dementia that has acceptable interpreting and reacting capabilities may benefit from making a wide variety of acoustic stimuli available to improve the stimuli-poor nursing home environment. For persons with reduced interpreting capabilities the sonic environment should be easy to disentangle. Explicit and clear meaning that may or may not relate to the past of the person should be beneficial. Not understanding the environment may lead to anxiety. These people may also lack the interpretation of time and space so sonic elements that help with understanding the day or the place may help. Persons with limited reacting capabilities may suffer from poor sonic environments while not being able to react appropriately. This may lead to behavior that is disturbing for other residents as well as for staff. Soundscapes may be more complex, diverse and challenging when interpretation capabilities are intact. The use of persona is motivated to account for a basic personalization of the resulting soundscape composition.

### 3.1. Designing for Effects

The evidence shows that an appropriate soundscape design could result in the following desired effects:

#### 3.1.1. Mood Changers

The potential of using sound for changing mood will be discussed within the framework of the circumplex model of mood [57,58]. As a depressed mood is common for residents with dementia, supporting them to increase valence and/or arousal using specific sounds is attractive. Based on the evidence for music therapy, the mood changers in soundscape design can be understood as a time-elongated session of music therapy, where the sounds are played in the sleeping room or the living room of the residents. As an additional benefit a resulting positive mood has been shown to broaden the scope of auditory attention [59], giving desired outcomes in improved social interaction. With respect to arousal, mood changers may support the diurnal pattern of activities in the nursing home and help to synchronize inhabitants with this pattern. At specific times of the day, engaging and activating sounds may be beneficial while during other periods calming sounds may be beneficial. Sounds with a low level of meaning that may not even attract attention but that show fast fluctuations especially in the higher frequency range (e.g., a morning bird chorus) typically increase arousal and could be used for example, in the morning. Lower frequencies and slow variations, on the other hand, tend to reduce arousal and have a calming effect. In addition, the meaning associated with foregrounded sounds and music can indirectly influence mood, yet mainly along the valence dimension. When used for persons with dementia, special care is needed. When using music, the effect of rhythm seems to remain longer than lyrics during progression of dementia. Yet melody recognition may still be useful. In view of reduced capabilities to disentangle sounds and predicting what comes, lower complexity sonic environments are beneficial. Pleasure and thus the increase of valence are more likely to be related to these easy-to-predict-sonic environments than in healthy persons. Using the meaning associated with sounds or music for people with dementia remains challenging. Although using sounds from their past seem attractive for creating arousal, care should be taken not to reduce valence through associations that may not be known by the soundscape designer (e.g., music heard frequently during war times). Yet, in any case, meaning should be clear. Sounds that can not easily be interpreted without an explicit context (e.g., waves breaking on the shore) should be avoided. As a good rule-of-thumb, any sound that is expected to trigger associations should have an obvious and immediate meaning to the designer even if reproduced in poor conditions (as a proxy for reduced hearing capabilities). Considering the expected outcome of these mood changers, one can think of reduced medication intake through engaging, calming or activating residents.

#### 3.1.2. Safety Enhancers

In order to enhance a safety feeling, basic stressors (like not knowing where one is and not knowing what time it is) need to be avoided while in line with the GUTS signals confirming safety should be provided continuously. Information about the current time and place is crucial with this respect. Auditory sources can give this information and can enhance the safety feeling in this way. As an example one can think of the church bells giving a specific sound each hour. Such a sound is easily recognized and incorporates the time information. The characteristics of the bell sound itself can even work as a spatial orientation sound, making it function as a soundmark in place and time orientation. Also in the framework of the GUTS sounds heard in the distance with a repeating character can confirm the presence of other people and the usual surrounding activity. Both biophonic and anthropogenic sounds could be used. This could be particularly important at the moment that the person with dementia is alone. Safety enhancers for people with dementia should be particularly clear and high fidelity sounds that can easily be recognized. Too much novelty should be avoided. Considering the expected outcome of these safety enhancers, one can think of more tranquil nights and better sleep quality (through relaxing and stabilizing a resident).

#### 3.1.3. Response Triggers

A sound can be used to evoke a (Pavlovian) reaction in a way that specific behavior is initiated. For this reactive purpose, clear and unique sounds are required, which in general generate low level associations in the brain and trigger an autonomous like response. They can also take part in the nomic or symbolic mapping as a level of understanding the environment. Following the daytime activity patterns and personal habits, such sound elements are in general present in a person’s nomic or symbolic map, as one can often relate specific sounds to the predicted and desired activity. In this way they arise from the life-long learning and experiencing of the temporal binding of daily sounds. In a home context, one can think of the typical sounds of cutlery or kitchen sounds as an indication that a meal is expected to follow. In the context of a nursing home, starting from the existing environment as experienced by a resident with dementia, amplification of nomic sounds and additional explicit symbols in the soundscape may be required. Considering the expected outcome of these these response triggers, one can think of enhanced efficiency through triggering, announcing and avoiding startle of a resident.

### 3.2. Soundscape Composition

**Figure 3:**
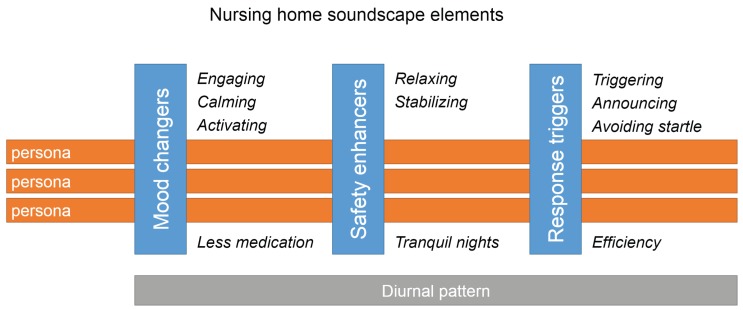
Representation of the nursing home soundscape design model, illustrating the 3 main behavior influencers with their expected outcomes, as scheduled over a diurnal pattern following the needs of a specific persona.

With the objective to obtain an appropriate soundscape, a daylong pattern of added sounds needs to be composed. These sounds consist of elementary acoustic stimuli and are intended to improve behavior to be in correspondence with the diurnal activities. As added sounds are used in the continuous (24 h a day, 7 days a week) soundscape intervention, the desired outcome will benefit from a high-quality acoustic comfort and from the maximal avoidance of disturbing sounds. As designed sound environment will continue in perpetuity, residents will become habituated to these sonic environments and this new norm will become the confirmation of safety. As learning is very limited in persons with dementia, habituation could be less than for healthy persons. Still, switching off any added sound should be done carefully and only if unavoidable as this change may trigger unwanted behavior. The healthy brain may contain multiple representations of the world around it and use parallel predictive models, yet it is unsure how strongly this ability is preserved in all forms of dementia. In the daylong sound pattern, as depicted in Figure 3, the different elementary acoustic stimuli (including silent periods), the levels of which are between the typical background and foreground levels, are scheduled appropriate to the diurnal pattern and the personal aspects (persona). The sounds that are foreseen should be dominant and attract attention only if they are intended to trigger certain behavior. Mood changers and safety enhancers should be easy to suppress or background. Furthermore, the design of the sonic environment is intended to amend or replace the sounds that are typical for the institutionalized environment with sounds that are typical for an everyday living environment, the selection of which is supposed not to give person specific adverse reactions as can be assured from prior personal information or from staff observation and monitoring. The added sounds will be designed to mask undesired unit sounds if these are likely to trigger an unwanted response and are not enhancing perceived safety. In other situations, the added sound will create additional context for the sounds that are already present, modifying their meaning. In the context of a nursing home the resulting sound pattern can be delivered to the sleeping room of a resident or to a living room common for all residents. Where the soundscape composition for a common living room needs to take into account the presence of multiple residents, the composition in case of an individual sleeping room can be targeted towards the specific resident. Preferred sounds may change over time, for example because of the progression of the disease. As persons with dementia are generally not able to express their like or dislike nor to modify the playback system, a suitable playback system will need to rely on an evaluation of the state of the resident by a care giver. In view of efficiency, the soundscape intervention technology may benefit from an easily accessible feedback system and from staff participation in the operational aspects of the deployment. The determination of the diurnal pattern and the selection of the specific acoustic stimuli can result from participation of staff and residents as can be obtained during co-creation sessions. Designing an improved sonic environment for persons with dementia requires not only sufficient knowledge of the everyday context and the state of the residents, but also thorough insight into acoustics and psychoacoustics. This publication could only provide some suggestions, yet involving an acoustical expert in training the staff responsible for tuning and maintaining the sound playback is crucial.

## 4. Discussion

In order to implement soundscapes for people with dementia, special requirements are needed to be fulfilled, in addition to basic standard guidelines.

As the intervention is intended to add acoustic stimuli to the existing soundscape, care should be taken that the resulting soundscape does not become chaotic. Sound levels of the added components need to be chosen in correspondence of the typical sound levels at the targeted places and with respect to the hearing capacity of a resident. The intention is to add sounds that mix in the level range between foreground and background levels. Indoor sound levels in nursing homes are reported for the different type of rooms present [10,60,61,62]. The existing soundscape in a nursing home (on the level of the living rooms) could often be described as giving an annoying, monotonous and uneventful perception [11]. This indicates that added acoustic stimuli could be deployed in these settings without obtaining a chaotic perception of the overall sonic environment.

In order to be effective, the deployment of the soundscape needs to take into account the foreground versus background characteristics and the timing aspects arising from diurnal temporal patterns. As a nursing home is an institution where staff is interacting with residents following their care needs, the anthropogenic soundscape component will follow the diurnal patterns arising from the organization of the care. In general, this follows a fixed daily pattern reflecting the different care and support activities (basic assistance such as helping patients bath, dress themselves, get up and down, walk, use of wheelchairs or walkers, food delivery, intake and administration of medication, support during social game sessions, …). Apart from the caregiver-resident interactions, the working schedule of the care givers with fixed moments of team shifts will also give rise to specific activity patterns. The diurnal composition of the delivered soundscape is beneficial through the support in the awareness of rhythms and routines of the residents, which is known to improve the care outcome [63].

**Figure 4:**
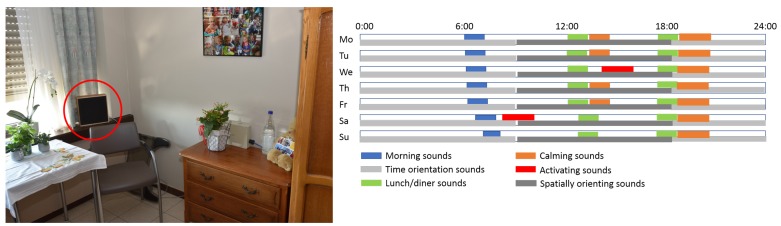
Illustration of a soundscape intervention: (**left**) photograph of a sleeping room setting with a soundscape player indicated with a red circle, (**right**) diurnal pattern of a composed soundscape with a weekly structure.

In previous work [64], a pilot soundscape intervention study was conducted to experiment with composed soundscapes (Figure 4). In order to obtain maximal desired outcome, a study of the level of acoustic comfort present in the different nursing homes was performed, consisting of reverberation time measurements and sound insulation measurements between rooms, initiating interventions that have been realized to improve the acoustic comfort level in specific rooms [65,66]. During these experiments, different acoustic stimuli were selected to be part of a soundscape composition that was played continuously in individual rooms of residents.The selection and timing of these stimuli resulted from co-creation sessions with staff and family members. Staff need to be included in design changes and environmental interventions because this will stimulate staff cooperation and improve resident care. The resulting selected acoustic stimuli consisted of Birdsong (bird signing in natural context, light natural sounds as background) and Wind (light breeze in natural context and sounds of leaves rustling in the trees) during the morning, Bell (bell of a church marking the hour), Cafeteria (sounds of people chatting and cutlery) around noon, Typewriter (sound of a person writing, using a typing machine and little bell) in the afternoon, Music (“Claire de lune”—Debussy) towards the evening and a Heartbeat (sound of heartbeat with a rate of approximately 60–80 bpm) in the evening. A first evaluation of the experiments (living labs) was based on a qualitative interview on the impression of experiences of the health care professionals who worked with the soundscapes. Globally, the soundscapes were experienced as positive; the impact on the ‘atmosphere’ was obvious and consequently also on the behavior of the persons with dementia, mainly due to the ‘orientating’ function of the soundscape and under the condition that the soundscape is tailored made. A certain degree of ‘habituation’ was observed but this was not necessarily considered negative.

**Figure 5:**
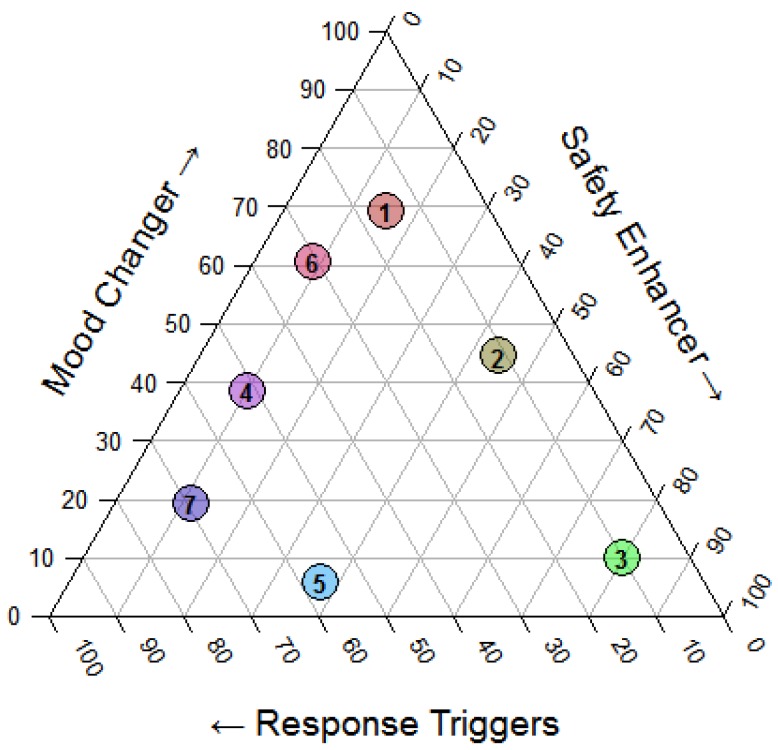
Ternary plot illustrating the mixing (relative importance indicated in percentages) of the mood, safety and response triggering aspects of the different acoustic stimuli: (1) Birdsong, (2) Wind, (3) Bell, (4) Cafetaria, (5) Typewriter, (6) Music, (7) Heartbeat as considered in a specific context. The saturation level of the color indicates the strength of the effects.

Where these sound tracks were selected during the co-creation sessions considering their appropriateness seen the timing characteristics and the specific nursing home context, they can also be considered in view of their supportive potential. Following the derived soundscape design framework a sound can contribute to the different desired effects in variable ways. This potential is illustrated in Figure 5 where the relative importance of the different desired effects (changing mood, stimulating the feeling of safety and triggering a response) is shown in a ternary plot, as resulted from a (subjective) single person assessment. The plot can be interpreted as a sound behavior influencing plot giving a visual interpretation of the behavior influencing effects of a sound in a specific context (specific place and time during the day) as is reflected in the ternary position and the influencing strength as is reflected in the saturation level of the color of the sound. A position more central in the triangle reflects a non-specific influencing sound, while a sound represented more to the corners indicates a sound with a specific influencing sound.

Taking into account the different possible deficits in the behavior pathway of a person with dementia, sounds should be intended to provide basic information in a redundant manner, as can be provided from both the foreground (meaning) pathway and the background (core affect) pathway. Considering that the different non musical sounds have a universal character and can be considered to have been present spread over a past lifetime, it can be expected that reminiscence [67], as originating from remaining long-term memory, can contribute in this way. The effect of ambient sound on the perceived safety was studied by Sayin et al. [68] and showed that when perceived social presence is higher and positive, the feeling-of-safety is also higher. In this view a typical bell sound of a church could act in this manner as it is intended to give an indication of an (active) community in the (perceived) neighborhood.

In understanding the role of sound on behavior, as is necessary in the design of a soundscape, one can also consider what is called a mind-state. Andringa et al. [69] described this approach as a model for describing the interaction between the core cognition and the peripheral sensing of a person. The interaction is described in a way that different arousal levels allow for different levels of mind-states. These levels range from a maximally restoring mind-state (sleep), over a restoring mind-state (characterized by fascination, automated tasks and automatic perception-action), an effortful mind-state (with novel tasks, directed attention and partial perception disengagement) to the highest arousal mind-state which is even effortful and even inefficient (from multitasking, directed attention switching and distractor claimed attention). Due to the cognitive deficits of persons with dementia, it can be expected from their activity levels that they mainly reside in a restoring mind-state. The use of an additional composed soundscape could help in stimulating and activating the residents towards a higher effortful mind-state.

Due to the burden of dementia, a detailed diagnosis is often not available, which hinders the detailed personalization of the approach. In order to overcome this problem, a practical methodology combining bedside information with specific investigations was introduced which can lead to a clinical outcome which offers a ground for an added level of personalization [51]. Based on the knowledge of cognitive relations, such outcomes could indicate desired sound management characteristics resulting in personalized sound stimuli to be included in the composition.

## 5. Conclusions

Based on a narrative review of existing knowledge on the influence of the sonic environment on well-being and behavior and on the deviant processing of acoustic stimuli by people with dementia, this publication proposes designing supportive soundscapes for nursing home residents with dementia and forwards an appropriate design methodology. In addition to a classical acoustic design-for-comfort that includes appropriate sound insulation of the facade and between rooms, adequate absorption for reducing unwanted sound as well as improving speech intelligibility and reducing installation and operational noise, soundscape design also includes the permanent playback (24 h/7 days) of a sound composition in different rooms of the nursing home. One of the main reasons for adding these sounds is to influence the behavior of residents, to result in a decreased BPSD. The permanent character of the optimized sonic environment and the ubiquitous presence of such an intervention can make it an attractive mode of therapy as compared to the more common music therapy. It could enlighten the existing nursing home soundscape which is often perceived as annoying, monotonous and uneventful. The suggested design methodology proposes reflecting on three types of influences that the sonic environment could have—changing mood, increasing perceived safety and triggering responses. Both clearly noticeable sounds (triggering responses, perceived safety) and subliminal backgrounds (mood changers, perceived safety) could be used for these purposes. In view of the burden of different cognitive deficits, which take a specific role in the interplay between perception, cognition and behavior of persons with dementia and which come on top of the changing auditory processing and cognition with age, a few guidelines are proposed to the soundscape designer concerning the choice of sounds in his or her composition for this specific target group. Reduced capability of auditory scene analysis, gating and object formation should be considered and therefore complex sound environments should be avoided. Associative memory deficits may result in unexpected behavioral responses and, hence, care should be taken when meaningful sounds are used as mood changers or safety enhancers. With reduced plasticity, new response triggers may take longer than expected to be learned and hence the designer may attempt to rely on common historical symbolic or nomic mapping of sounds. As illustrated in the narrative review, dementia may take several forms some of which result in hefty and very different responses to sound ranging from sound aversion to musicophilia. Together, with the different meaning that could be given to sounds within the context of life long experience, this calls for a personalized approach to soundscape design. Co-creation involving care givers and persons from the direct surrounding (e.g., family) of the patient could be a beneficial in this respect. Technology could help by providing suitable interfaces to sound databases as well as opportunities for feedback. It is expected that an appropriate application of these ideas to the design of the living environment of nursing homes for people with dementia could reduce the use of pharmaceuticals and improve the working conditions for the staff in addition to improving the well-being of the residents.
